# A Role for Excitatory Amino Acids in Diabetic Eye Disease

**DOI:** 10.1155/2007/36150

**Published:** 2007-06-25

**Authors:** Jose E. Pulido, Jose S. Pulido, Jay C. Erie, Jorge Arroyo, Kurt Bertram, Miao-Jen Lu, Scott A. Shippy

**Affiliations:** ^1^Department of Ophthalmology, Mayo Clinic, Rochester, MN 55905, USA.; ^2^Division of Ophthalmology, Beth Israel Deaconess Medical Center, Harvard Medical School, Boston, MA 02215, USA.; ^3^Department of Chemistry, The University of Illinois at Chicago, Chicago, IL 60607, USA.

## Abstract

Diabetic retinopathy is a leading cause of vision loss. The primary clinical hallmarks are vascular changes that appear to contribute to the loss of sight. In a number of neurodegenerative disorders there is an appreciation that increased levels of excitatory amino acids are excitotoxic. The primary amino acid responsible appears to be the neurotransmitter glutamate. This review examines the nature of glutamatergic signaling at the retina and the growing evidence from clinical and animal model studies that glutamate may be playing similar excitotoxic roles at the diabetic retina.

## 1. INTRODUCTION

Diabetic retinopathy causes 12,000–24,000 new cases of blindness each year in the United States alone and is the leading cause of blindness in persons between the ages of 20–74 [1]. The incidence is increasing as the number of persons with diabetes mellitus rises. In 2005, it was estimated that 7% of the population in the United States, 20.8 million people, had diabetes mellitus [1]. Diabetic retinopathy is a major cause of disability with an estimated 40% of adults over the age of 40 years having some form of diabetic retinopathy and 8% having vision-impairing diabetic retinopathy [2]. The prevalence of diabetic retinopathy differs with racial characteristics with 50% of adult Hispanics with diabetes mellitus having some form of diabetic retinopathy [3].

Risk factors for the development of diabetic retinopathy have been well described and can be divided into systemic factors and local factors. The systemic factors include duration of diabetes, severity of diabetes as measured by hemoglobin A1c, hypertension, anemia, renal disease, and lipid levels [4]. The local protective factors include myopia, the presence of chorioretinal scars, and optic atrophy while local aggravating factors include inflammation and prior ischemia [5, 6]. Diabetic retinopathy is caused by an ischemic microvasculopathy and it is divided into nonproliferative and proliferative forms [7]. Both of these forms are presaged on damage to the capillaries and then the secondary response to the damage. In the nonproliferative form, there is evidence of leakage from the capillaries as well as drop out of capillaries. The leakage is manifest as swelling of the retina and deposition of lipoproteinaceous material in the retina (hard exudates) as well as microaneurysmal sacculations of the capillaries and intraretinal hemorrhages [8]. Following the loss of capillaries, there is a hypoxic response by the retina with release of vascular endothelial growth factor (VEGF) [9]. VEGF is in part what causes leakage from the remaining capillaries. It is important also to note that the normal retinal circulation is under autoregulation [10]. There is a compensatory increase or decrease in flow in the retinal circulation depending upon the physiologic demands from the retina. This autoregulation may be driven by local nitric oxide production [11]. What drives the autoregulation is at the present time speculative but may be intrinsically related to the excitatory amino acids because nitric oxide is associated with excitatory amino acids as will be discussed later [12]. Another aspect of diabetic retinopathy that is poorly recognized and occurs because of autoregulation is a compensatory increase in the flow through the remaining vessels [10].

With ischemia, there is a significant release of VEGF, which causes secondary growth of neovascular tissue on the surface of the retina. This neovascular tissue is comprised of very immature vessels that leaks further and bleeds readily. This is an important cause of visual loss. Ultimately, the vessels cause a secondary fibrotic response as well and this causes scarring on the surface of the retina.

Besides VEGF, there may be a panoply of other factors that may be associated with the changes noted. These factors may be proteins, peptides, and small molecules [13]. There is a marked increase in the number of proteins seen in the vitreous in both experimental as well as in clinical diabetic retinopathy [13, 14].

The vitreous is also affected by diabetes and the changed vitreous is involved in the development of diabetic retinop athy [15, 16]. The vitreous contracts probably because of nonenzymatic glycosylation [17]. The contraction of the vitreous then allows growth of the neovascular tissue onto its posterior surface and also causes the tissue to bleed.

## 2. EXCITATORY AMINO ACIDS

Amino acids or their metabolic products have been shown to be neurotransmitters [18]. Olney was the first to recognize that a group of these amino acids were excitatory [19, 20]. He labelled them as excitatory because the released amino acids cause rapid depolarization of glutamate sensitive cells. The number of amino acids that have been designated as excitatory has grown since Olney's initial studies and include glutamate, glycine, aspartate. Glutamate is a critical excitatory amino acid in the brain and the most important excitatory amino acid in the retina.

The entries in Table 1 show the types and diversity of glutamate receptors. There are two classes of glutamate receptors, ionotropic and metabotropic. The ionotropic receptors work via ion channels. The metabotropic receptors are G-protein coupled receptors. There are three subclasses of ionotropic receptors: N-methyl-D-aspartate (NMDA), amino-3-hydroxy-5-methyl-4-isoxazolepropionate (AMPA), and kainate type receptors. The NMDA receptors are the ones that are most associated with excitatory neurotoxicity and calcium entry into the cells. The calcium entry causes release of caspases from the mitochondria leading to apoptosis. NMDA receptors are made up of 3 different subunits, NR1, NR2A-D, and, in some cases, NR3A or B subunits. The receptor is probably composed of a tetramer of these subunits. Alternative splicing further helps in adding pharmacologic differences to the action of the receptors. There is a diversity of NMDA receptor types in different regions of the central nervous system.

There are at least eight metabotropic glutamate receptors (mGluR). These are subdivided into three subclasses. Type I metabotropic receptors are associated with intracellular phosphotidyl inositol metabolism. Type II and III receptors are associated with an inhibitory cAMP cascade as well as other postsynaptic cascades that lead to the release of Ca^2+^ from intracellular stores. There is some data to suggest that some of the type II mGluRs are neuroprotective.

There is a relationship between the metabolism of glutamate, glutamine, and GABA. GABA is a synthesized in the presynatic axons of certain neurons via the use of glutamate decarboxylase. It is the major inhibitory neurotransmitter in the brain and retina.

**Figure 1:**
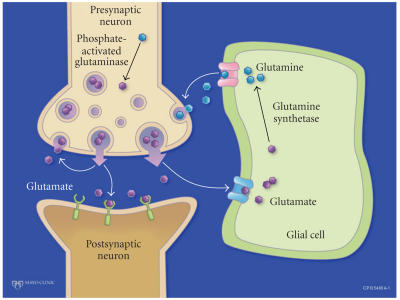
Glutamate cycle in the synaptic space.

Because of the significant neurotoxic effects of glutamate, glutamate concentrations have to be very closely regulated in the synapse. Much of the released glutamate is taken up by the surrounding glial cells and converted into glutamine. The glutamine is then taken up by the presynaptic axon. Glutamine is deaminated and turned back into glutamate (Figure 1). Direct glutamate reuptake by the presynaptic neuron accounts for a small amount of the released glutamate. Another small amount actually escapes from the synaptic space and may have significant peripheral effects [21]. The amount that escapes appears to increase in pathologic conditions.

For glutamate, there are five known high-affinity excitatory amino acid transporters: EAAT1 (GLAST), EAAT2 (GLT-1), EAAT3 (EAAC1), EAAT4, and EAAT5 [22–26]. The transporters and their predominant locations are shown in Table 2. Uptake of glutamate into astrocytes is mediated by GLAST (also found in Müller cells) and GLT1 (or EAAT1 and 2) and into neurons by EAAC1 (or EAAT3), EAAT4, and EAAT5, of which the last primarily is found in the retinal photoreceptor cells. In addition, there are glutamine transporters that need to be synchronized to transport glutamine from the astrocytes into the neurons (Figure 1).

### 2.1. Excitatory amino acid and disease states

Recently, there has been a growing appreciation that disruption of this cycle leads to glutamate levels that may fall outside normal ranges and lead to tissue dysfunction and neuronal death [27]. This process has been implicated in hepatic failure-associated CNS dysfunction [28], HIV-associated dementia [29], ischemia [30], Alzheimer's [31], and Huntington's disease [32]. The detrimental effects of excess glutamate has largely been related to iontropic receptor overactivation [27, 30–35]. Treatments that have been proposed and tested then are aimed at interfering with these receptors though there is now data that implicates the metobotropic receptors for damage to the postsynaptic cells as well [27, 31–33]. With hepatic failure, there is an increase in ammonia. The ammonia increase causes a downregulation of GLT1 causing an accumulation of extracellular excitatory glutamate. This is thought to be one of the leading causes of the CNS derangements seen with hepatic failure [28]. Following ischemic stroke, there is a marked increase in extracellular glutamate as well [36]. Both clinical and animal studies have demonstrated an association between the increase in glutamate levels with worsening neurological deficits. How the increase of glutamate occurs, whether it is excessive release, poor reuptake or a problem in glutamate-glutamine cycling is still unknown. Hypoglycemia has also been associated with glutamate neurotoxicity in the central nervous system. Hypoglycemia has also been associated with glutamate neurotoxicity in the central nervous system [37]. This same neurotoxicity has also been reproduced by using iodoacetate which inhibits glycolysis. The neurotoxicity induced by hypoglycemia is inhibited by both glutamate inhibitors as well as pyruvate presumably by allowing progression of oxidative phosphorylation [38]. In vivo, potentiation of glutamate-mediated neuronal damage after chronic administration of the glycolysis inhibitor iodoacetate [38]. Hyperglycemia has not been associated with glutamate neurotoxicity in the central nervous system.

### 2.2. NMDA antagonists

MK-801 was the first NMDA antagonist that was used but clinically it was associated with coma and delirium. Amantadine, which was originally developed to interfere with influenza virus uptake and was then used in patients with Parkinson's disease, was noted to have NMDA receptor antagonism. Memantine, a derivative of amantidine, is a more efficacious NMDA receptor antagonist and has been approved for the treatment of Alzheimer's disease and is being investigated for possible use in other diseases including vascular dementia, neuropathic pain, and glaucoma [27].

### 2.3. Excitatory amino acids and the retina

The initial studies by Dreyer showing that glutamate was elevated in glaucoma were subsequently brought into question [39]. Subsequent studies by ourselves in this issue and others have shown that glutamate is elevated in the vitreous in glaucoma, diabetes mellitus, and retinal detachments and thus, similar to ischemic brain injury, glutamate may play an important role in these diseases [40–42].

Among the five excitatory amino acid transporters identified to date, four are found in the retina. EAAT1 (GLAST) is found in Müller cells and astrocytes [43]. EAAT2 (GLT1) is localized to cones and two types of bipolar cells [44]. EAAT3 (EAAC1) is found on horizontal, amacrine, and ganglion cells, and occasionally on bipolar cells [45]. EAAT5 is localized to photoreceptors and bipolar cells [46].

Glutamate receptors are present throughout the retina. The Müller cells in the retina act as the astrocytes in the brain. They have GLAST expression and are important in removing glutamate from the synaptic space of the retina [47]. GLAST1a is also expressed. In this form, exon 3 is not expressed which decreases the efficacy of glutamate uptake. This may be a method of regulating the efficacy of Müller cell glutamate uptake [48].

Bipolar cells have kainate, AMPA receptors, and NMDA receptors [21, 49]. For all bipolar cell types, the AMPA receptor subunits GluR2, 2/3, and 4 are the most common types while GluR1 are rare. The kainate GluR6/7 are predominantly associated with diffuse bipolar (DB6) and rod bipolar cells. The NMDA receptor, NR1C2, is seen in flat midget and DB3 axons. The kainate receptors are seen primarily in dendrites of off-bipolar cells associated with cone axons [21]. Horizontal receptors also have AMPA type receptors [50]. On-bipolar cells appear to use metabotropic receptors [51]. In the inner retina, glutamate receptors are present in the ganglion dendrites and amacrine cells. Metabotropic type II receptors are present on certain amacrine cells [52].

### 2.4. Retinal diseases associated with elevated glutamate levels in the vitreous

#### 2.4.1. Glaucoma

It has been suggested that increased extracellular glutamate levels may be due, at least in part, to a failure of glutamate transporter buffering [53]. The data has to be carefully evaluated to look at the acute changes and the chronic changes following injury. For instance, following crush injury to the optic nerve, there appears to be an acute increase in GLT1 expression followed by a decrease to near normal levels over time [54].

In a chronic glaucoma model, both ionotropic and metabotropic antagonists appeared to be effective in limiting damage from glaucoma [55]. The NMDA antagonists appeared to have a greater effect in limiting damage than the metabotropic antagonists but this study shows that metabotropic receptors may be important in glutamate-induced damage as well. Previous studies using immunohistochemistry have demonstrated a reduced expression of EAAT1 in a rat glaucoma model [56]. It has been shown in rats that treatment with antisense-oligonucleotides to GLT-1 leads to increased vitreous glutamate levels and RGC death [33]. In addition, in human glaucoma the expression of this glutamate transporter is reduced at the protein level [57]. Following experimental glaucoma induction in rats, with subsequent optic nerve damage, Martin et al. [56] recently found no change in the expression of GLT1 by immunohistochemistry. However, Western blot analysis revealed a significant decrease in the levels of GLT1 protein. Interestingly, following optic nerve transsection, which leads to extensive RGC death, the GLT1 protein levels were found to be increased in this study [56].

As mentioned, the relationship between glaucoma and glutamate has been questioned. In fact, a recent article showed no relationship between glutatmate levels and glaucoma in persons with glaucoma requiring vitrectomy [58]. This is different from animal models and further human studies are required.

#### 2.4.2. Retinal detachments and diabetic retinopathy

In clinical studies, there are mildly elevated levels of glutamate in the vitreous with rhegmatogenous retinal detachments [41] and markedly elevated in cases with diabetic retinopathy [40] though the number of studies is very limited and further studies are definitely warranted to confirm these findings. The levels of glutamate in the vitreous of patients with rhegmatogenous retinal detachment not associated with diabetic retinopathy was 25% higher than the glutamate levels in the vitreous of patients who underwent vitrectomies for other causes [59]. Rhegamtogenous retinal detachments are associated with mild retinal ischemia since neovascularization is rarely seen with rhegmatogenous retinal detachments and that may be the reason the glutamate levels were slightly elevated. The cases of diabetic retinopathy that have been associated with elevated glutamate levels were cases with proliferative diabetic retinopathy. In the study, Ambati and colleagues evaluated levels of glutamate in eyes with proliferative diabetic retinopathy and compared the levels to controls who underwent vitrectomies for other causes. They controlled for hemorrhage in the eyes with proliferative diabetic retinopathy by determining hemoglobin levels. Even controlling for elevated hemoglobin levels in the vitreous (as a marker for vitreous hemorrhage), the glutamate levels were at least twice as high as control eyes. Deng et al. also found elevated vitreous levels of glutamate in cases of proliferative diabetic retinopathy [60]. In this journal, our group using capillary electrophoresis, a low volume-sensitive method reports elevated levels of glutamate as well. On the contrary, Asensio Sanchez and his coauthors did not find elevated glutamate levels in the vitreous of patients with diabetes in comparison to their control patients [61]. Overall, there are more studies showing elevation in glutamate levels with proliferative diabetic retinopathy than studies showing no differences but further studies are needed in humans and if they do show elevations, studies to determine the use of glutamate inhibitors may then be warranted.

Interestingly, even in animal models of diabetes, the determination of levels of glutamate in the eye is conflicting. In a study by Obrosova et al., that looked at retinas removed from STZ-induced diabetes within 6 weeks following induction of diabetes, showed that the levels of glutamate in the retinas were similar to those in the control eyes for diabetic mice while they were reduced compared to control eyes for diabetic rats [62]. In this study, they only determined the intraretinal levels and not the vitreous levels. Ward et al., in a similar animal model showed no difference in glutamate metabolism in the retinas [63]. Conversely, other studies have shown that glutamate levels are increased and it appears that glial cell metabolism is affected [34, 64]. Kerns showed that in their model of alloxan-induced diabetic rats, the glutamate levels in the retina were significantly elevated by 40% compared to control retinas after two months of hyperglycemia [65]. It may be that compared to the Obrosova study which did similar intraretinal glutamate sensing, the difference in comparing to normal after an extra 2.5 weeks of significant hyperglycemia in the Kerns study may have been the reason for Kerns finding a difference in glutamate levels while Obrosova did not. Lieth et al. showed that in ex vivo retinas of STZ-induced diabetes there was reduced glutamate oxidation by 38% compared to controls. Similarly, Puro used freshly dissected Müller cells from normal rat eyes and cells from STZ-induced diabetes eyes to demonstrate a statistically significant diminution in the glutamate transport in the diabetic group after four weeks of diabetes. After four weeks of diabetes, a statistically significant diminution could be seen in the glutamate transporter between these two groups. By 13 weeks, there was a 67% decrease in transporter function compared to controls. This is similar to other causes of retinal ischemia, where the GLAST function in the Müller cells is markedly diminished [66]. In addition, neural cells may have altered glutamate receptor function and calcium metabolism [67].

Besides direct neurotoxic effects, elevated glutamate levels may have other indirect retinotoxic effects as well. We have previously shown that nitrates are increased in diabetic retinopathy [68]. Glutamate elevation is associated with nitric oxide but how it is related is not completely understood. Kerns in his study using rats following 2 month of hyperglycemia showed that inhibiting glutamate levels decreased NO oxide production [65]. Subsequently, no other studies have been done to evaluate this. Considering that nitric oxide appears to be an important regulator of retinal vasculature autoregulation, further studies to understand the relationship between nitric oxide levels and glutamate are warranted. Finally, there are other pathways that are affected by excitotoxicity. Protein Kinase C (PKC) is activated by NMDA activation. PKC-*ζ* inhibitors prevent some of the damage from NMDA activation but PKC-*β* inhibitors do not inhibit neuronal death. PKC-*β* is activated in diabetes and appears to be related to diabetic retinopathy but the relationship between PKC-*ζ* and diabetes has not been established though it is interesting to note that PKC-*ζ* has been associated with upregulation of VEGF in other systems [69, 70].

## 3. SUMMARY

There appears to be a relationship between diabetic retinopathy and elevated glutamate levels similar to other cases of CNS ischemia and glaucoma, however, further studies are required to confirm this. Though part appears to be an effect of ischemia on the function of Müller cells, the exact pathophysiology of how diabetes causes elevated glutamate levels in the vitreous also has to be determined. Whether glutamate elevation is just a marker disease or actually adds to the complications of diabetes has not been established but from other diseases that cause elevation of glutamate in the CNS, treatment by glutamate inhibitors appears to decrease neurotoxicity. Results of clinical trials of memantine and glaucoma are ongoing and will help to determine the importance of glutamate in ocular pathologies. How glutamate affects other important factors, for instance nitric oxide and VEGF, have to be further elucidated.

## Figures and Tables

**Table 1:** Glutamate Receptors.

		Isoforms	Glutamate Response

**Ionotropic**	NMDA	NR1, NR2A-D and NR3A-B	Increase Ca^2+^/Na^+^ intracellular influx
	AMPA	GluR1, GluR2, GluR3, and GluR4	Increase Na^+^/(Ca^2+^) intracellular influx
	Kainate	GluR5, GluR6, GluR7, KA1, and KA2	Increase Na^+^/(Ca^2+^) intracellular influx

**Metabotropic**	Type I	mGluR1, mGluR5	Increase intracellular inositol phosphate and diacylglycerol
	Type II	mGluR2, mGluR3	Decrease intracellular cyclic adenosine monophosphate
	Type III	mGluR4, mGluR6, mGluR7, and mGluR8	Modulate intracellular cyclic adenosine monophosphate

**Table 2:** Glutamate Transport Proteins.

		Predominant Retinal Localization

**Glial**	EAAT1, GLAST	Müller cells and astroglia
	EAAT2, GLT1	Bipolar and cone cells

**Neuronal**	EAAT3, EAAC1	Horizontal, amacrine, ganglion, and bipolar cells
	EAAT4
	EAAT5	Photoreceptor and bipolar cells
